# How do patients sleep after orthopaedic surgery? Changes in objective sleep parameters and pain in hospitalized patients undergoing hip and knee arthroplasty

**DOI:** 10.1007/s00264-023-05862-2

**Published:** 2023-06-10

**Authors:** Jacopo Antonino Vitale, Giuseppe Banfi, Marco Viganò, Francesco Negrini

**Affiliations:** 1 Schulthess Klinik, Zürich, Switzerland; 2 IRCCS Istituto Ortopedico Galeazzi, Milan, Italy; 3 Vita-Salute San Raffaele University, Milan, Italy; 4 Istituti Clinici Scientifici Maugeri IRCCS, Tradate, Varese, Italy; 5 Department of Biotechnology and Life Sciences, University of Insubria, Varese, Italy

**Keywords:** Sleep, Actigraphy, Knee arthroplasty, Hip arthroplasty, Pain, Rehabilitation, Recovery

## Abstract

**Purpose:**

The aim of this observational cohort study was to assess actigraphy-based sleep characteristics and pain scores in patients undergoing knee or hip joint replacement and hospitalized for ten days after surgery.

**Methods:**

*N*=20 subjects (mean age: 64.0±10.39 years old) wore the Actiwatch 2 actigraph (Philips Respironics, USA) to record sleep parameters for 11 consecutive days. Subjective scores of pain, by a visual analog scale (VAS), were constantly monitored and the following evaluation time points were considered for the analysis: pre-surgery (PRE), the first (POST1), the fourth (POST4), and the tenth day (POST10) after surgery.

**Results:**

Sleep quantity and timing parameters did not differ from PRE to POST10, during the hospitalization whereas sleep efficiency and immobility time significantly decreased at POST1 compared to PRE by 10.8% (*p*=0.003; ES: 0.9, moderate) and 9.4% (*p*=0.005; ES: 0.86, moderate) respectively, and sleep latency increased by 18.7 min (+320%) at POST1 compared to PRE (*p*=0.046; ES: 0.70, moderate). Overall, all sleep quality parameters showed a trend of constant improvement from POST1 to POST10. VAS scores were higher in the first day post-surgery (4.58 ± 2.46; *p*=0.0011 and ES: 1.40, large) compared to POST10 (1.68 ± 1.58). During the time, mean VAS showed significant negative correlations with mean sleep efficiency (*r* = −0.71; *p*=0.021).

**Conclusion:**

Sleep quantity and timing parameters were stable during the entire hospitalization whereas sleep quality parameters significantly worsened the first night after surgery compared to the pre-surgery night. High scores of pain were associated with lower overall sleep quality.

## Introduction

The correct expression of circadian rhythmicity is fundamental for the body homeostasis and humans perform optimally when all biological rhythms, including sleep-wake cycle, are in sync [1, 2]. Sleep is a basic requirement for human health and serves important psychological and physiological functions [3]. As recommended by the National Sleep Foundation [4], adults should obtain at least seven to nine h of sleep per night to maintain optimal health and functioning and it has been shown that the absence of adequate sleep is linked to a large number of adverse outcomes, including impairments to cognitive performance [5], mood [6], and appetite regulation [7], as well as critical metabolic [8] and immunologic processes [9].

Orthopaedic surgery is an event that has the potentiality to disrupt circadian rhythm and impair sleep quantity and quality. Actually, joint arthroplasty is considered the gold standard for severe knee and hip osteoarthritis, a pathology that has been estimated affects more than 240 million people worldwide [10–13]. Sleep impairment after hip and knee arthroplasty is multifactorial and still poorly understood. First of all, post-operative pain is a potential cause of sleep deprivation or disturbances. In one post-total hip arthroplasty study, 52% of patients woke up due to moderate to severe pain on the first postoperative night [14, 15]. At the same time, pain perception is increased by poor sleep quality, leading to a potential vicious cycle, in which pain decreases sleep quality, while simultaneously impairment of sleep quality increases pain perception [16, 17]. Further, also preoperative sleep quality impairment can lead to increased post-operative pain and painkillers consumption, underlying the complex and bidirectional interaction between sleep and pain [18]. In order to manage post-operative pain, painkillers are regularly administered after surgery. However, opioids, drugs commonly used for post-operative pain [19], are linked to modification in sleep architecture and they interfere with sleep quality [20]. Anesthesia is another crucial aspect that can disrupt sleep in post-operative patients. Both general and spinal anaesthesia, commonly performed for joint arthroplasty, have the potential to decrease sleep quantity and quality; however, spinal anaesthesia has been seen to impact less in the immediate post-operative period on the sleep of the patient [21]. While all the aforementioned aspects concur to disturb sleep particularly in joint surgery, sleep quality and quantity may be also influenced by other exogenous and endogenous factors such as primary sleep disorders, gender, anxiety, or bedroom environment [22, 23].

Post-operative period in joint arthroplasty is of the utmost importance because of the need of early rehabilitation. It has been shown that starting as early as the first post-operative day rehabilitation decreases hospital length of stay and overall costs of the procedure, without increasing number of adverse reaction [24, 25]. In order to perform rehabilitation, it is important that patients could gather as much strength and motivation as possible [26, 27]. The correct physiology of night-time sleep could potentially enhance patients’ collaboration and internal motivation, ultimately increasing the overall quality of rehabilitation and preventing the transient cognitive deficit linked to sleep deprivation.

Overall, reducing the incidence of sleep disturbance has the potential to decrease pain and enhance the patient’s mental status during daytime hours after joint arthroplasty, which may improve functional outcomes and hasten postoperative recovery. Inversely, persistent sleep disturbance after hip or knee surgery may increase pain and jeopardize the postoperative course while straining the physician–patient relationship [28]. To the best of our knowledge, no previous study objectively examined sleep changes in hospitalized patients during the inpatient rehabilitation period after orthopedic surgery. Therefore, the aim of this study was to assess actigraphy-based sleep characteristics and pain scores in patients undergoing knee or hip joint replacement and hospitalized for 10 days after surgery. We hypothesized to detect a worsening in sleep quality together with an increase in pain scores, immediately after surgery and to observe an improvement of these variables in the last days of hospitalization.

## Materials and methods

### Study design

The study protocol was approved by the Institutional Ethics Review Committee of Vita-Salute San Raffaele University (Milan, Italy; protocol number: 102/INT/2018) in compliance with the current national and international laws and regulations governing the use of human subjects (Declaration of Helsinki II). This trial was registered to the ClinicalTrials.gov registry (NCT03572920). The subjects were informed about the potential risks and benefits of the study before the start of the experimental procedures and written informed consent was obtained from all participants. This observational cohort study was conducted in accordance with the STROBE guidelines [29] between December 2018 and June 2019 at the IRCCS Istituto Ortopedico Galeazzi (Milan, Italy).

### Study participants

**Fig. 1:**
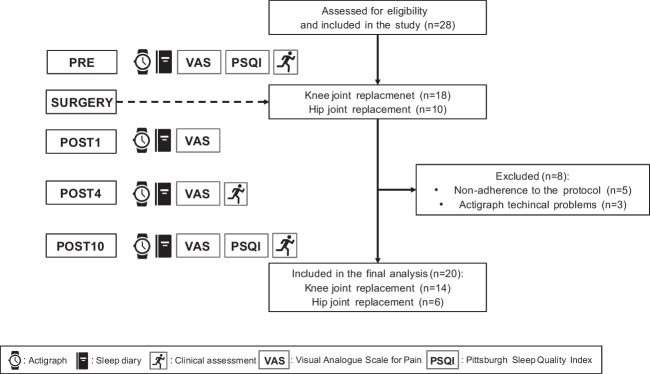
Flow chart of the study

The inclusion criteria for the study were as follows: scheduled hip or knee surgery; hospitalization and execution of the rehabilitation program, inside the hospital, for ten consecutive days after surgery; age between 50 and 75 years old. Exclusion criteria were as follows: body mass index (BMI) ≤18.5 or ≥40.0; mental or neuromuscular disorders; regular melatonin consumption during the preoperative period; and the presence of clinically diagnosed sleep disorders. All subjects were screened by a physiatrist for eligibility. The experimental procedures lasted 11 consecutive days: starting the day before surgery and ending the tenth day after surgery. Subjects’ sleep through actigraphy and subjective scores of pain, by a visual analog scale (VAS), was constantly monitored for the entire experimental protocol. For the purposes of the study, the following evaluation time points have been considered: pre-surgery (PRE), the first (POST1), the fourth (POST4), and the tenth day (POST10) after surgery. In addition, subjects completed the Pittsburgh Sleep Quality Index (PSQI; [30] at PRE and POST10 and four functional tests (see “*Clinical and functional tests*” for details) were performed only in three time points (PRE, POST4, and POST 10) since no data was collected at POST1 for clinical reasons (i.e., impossible tests execution the day after surgery). The study flow chart is shown in Fig. 1.

### Power analysis

Based on the limited existing literature (Manning et al., 2017; Myoji et al., 2015), it was considered clinically significant to observe a mean effect size of 0.80 in sleep quality between pre- and post-surgery. Therefore, considering an α level with *p*=0.05 and a power of 85%, it was estimated that 17 subjects would be needed in order to detect a statistically significant difference in sleep efficiency. To be more conservative and to prevent a possible drop of subjects during the study (estimated in 10–15%), the sample size has been increased to a total of 20 subjects. The sample size calculation was performed with the use of the G-Power software (G-Power version 3.1, Düsseldorf, Germany).

### Surgical and rehabilitation procedures

We selected patients suffering from severe knee or hip osteoarthritis, with orthopedic prescription of either elective hip or knee joint replacement. In case of patients suffering from hip osteoarthritis, THA was carried out following a mini-invasive postero-lateral approach [31]. As far as patients suffering from knee osteoarthritis are concerned, the type of arthroplasty was prescribed by the referent orthopaedic surgeon depending on the clinical and radiographic characteristics of the patients: if osteoarthritis was generalized and affected both medial and lateral segment of the knee, total knee joint replacement was performed; if osteoarthritis affected only medial or lateral compartment of the knee, unicompartmental knee joint replacement was performed, to potentially reduce both surgery time and increase recovery time [32]. All surgeries were performed under spinal anaesthesia.

The rehabilitation protocol started as early as the first post-operative day in the orthopaedic surgery ward, when patients were already verticalized if possible. In the first two days after surgery, patients followed a rehabilitation protocol, led by physical therapists 30 min/day. In the third post-operative day, the patients were moved in the rehabilitation ward and rehabilitation time was increased to 70 min/day. The main goal of the rehabilitation treatment was to regain functional independence as soon as possible. To pursue this aim, specific physical exercises were performed: passive and active mobilization of the involved joint, strengthening and proprioceptive training of the lower limbs, gait training using two crutches. During rehabilitation, an opioid-sparing analgesic protocol was followed. In detail, opioids and paracetamol were administered for the first three days after surgery to all subjects whereas NSAIDs were administered only when needed (i.e., in 12 subjects, the 60% of total sample).

### Sleep assessment

All subjects wore the Actiwatch 2 actigraph (Philips Respironics, Portland, OR) to record sleep parameters for 11 consecutive days during hospitalization, from PRE to POST10. A sensitivity threshold of 40 activity counts was utilized to detect sleep parameters [33]. Each subject completed daily a sleep diary to record bedtime (the self-reported time at which a participant went to bed attempting to sleep), get-up time (the self-reported time at which a participant got out of bed and stopped attempting to sleep), the number of nocturnal awakenings, and the minutes spent without wearing the actigraph. Data derived from the sleep diaries and wrist activity monitors were used to determine patients’ sleep parameters. In details, nine sleep parameters were measured: (1) sleep onset (Son), the time at which a participant first fell asleep after going to bed; (2) sleep offset (Sof), the time at which a participant last woke before getting up; (3) time in bed (TB), the amount of time spent in bed attempting to sleep between bedtime and get-up time; (4) total sleep time (TST), the number of minutes of sleep obtained during a sleep period; (5) sleep efficiency (SE), the percentage of time in bed actually spent sleeping; (6) sleep latency (SL), the period of time between bedtime and sleep onset time; (7) wake after sleep onset (WASO), the amount of time spent awake after sleep has been initiated; (8) immobility time (IT), the total time, expressed in percentage, spent without recording any movement during time in bed; (9) fragmentation index (FI), the sum of the time spent moving and the immobility phases of one min, both expressed in percentage, divided by the number of immobility phases.

### Pittsburgh Sleep Quality Index

The PSQI is the most commonly used subjective measure of sleep dysfunction [30]. The PSQI is a 19-item self-report questionnaire assessing sleep quality and sleep disorders and results in a total global score from 0 to 21 (7 items with a response scale ranging from 0 to 3) where the lower scores indicate the better sleep quality. PSQI was administered at PRE and POST10, before discharge.

### Visual Analog Scale for pain

Subjective ratings of pain were assessed all days, from PRE to POST10, using a 10-cm visual analog scale anchored by two verbal descriptors, one for each symptom extreme [34]. VAS data were collected at the same time of day. Subjects indicated their subjective ratings of pain, which ranged from “no pain at all” (score of 0) to “the worst imaginable pain” (score of 10) [35].

### Clinical tests

All patients underwent at PRE, POST4, and POST10 the following clinical evaluations: (1) the Ten-Meters Walking Test (10MWT) to measure the self-paced walking speed of patients [36]; (2) (TUG) to examine balance, posture transitions, and change of direction [37]; (3) the Functional Independent Measure (FIM), a scale to investigate the autonomy of patients during everyday life [38]; and (4) the Barthel Index (BI) to evaluate the time and amount of actual physical assistance required by patients to perform the activities of daily living [39, 40].

### Statistical analysis

#### Sleep parameters

The actigraphy-based sleep parameters are expressed as the mean ± SD. Each sleep parameter was calculated four times (PRE, POST1, POST4, and POST10). The normality of the distribution of each sleep parameter was then checked four times using graphical methods and Shapiro–Wilk’s test. TIB, TST, IT, and FI showed a normal distribution and, to test the null hypothesis of no differences among PRE, POST1, POST4, and POST10, a repeated-measures one-way analysis of variance (RM-ANOVA) followed by the Tukey-Kramer post hoc tests was applied. On the contrary, the remaining sleep parameters had no normal distribution and were treated with nonparametric Friedman test followed by Dunn’s multiple comparisons. In addition, PSQI displayed a normal distribution and a paired Student *t*-test was utilized to compare the results between PRE and POST10. A *p*-value ≤ 0.05 was considered statistically significant.

#### Clinical outcomes

Normal distribution of VAS was tested using graphical methods and Shapiro–Wilk’s test four times (PRE, POST1, POST4, and POST10) while FIM, BI, 10MWT, and TUG were tested only three times since no data was collected at POST1. None of the parameters showed a normal distribution; therefore, a Friedman test followed by Dunn’s multiple comparisons was applied. All clinical variables were expressed as the mean ± SD and a *p*-value ≤ 0.05 was considered significant.

#### Effect size

Partial eta-squared (*η*^2^_p_) was used to determine the magnitude of the effect for all significant outcomes in RM-ANOVA using the small (<0.13), medium (0.13–0.25), and large (>0.25) interpretation for effect size [41] while effect sizes (ES) for pairwise comparison were calculated using Cohen’s *d* and considered to be either trivial (effect size: <0.20), small (0.21–0.60), moderate (0.61–1.20), large (1.21–2.00), or very large (>2.00) [42].

#### Correlation analysis

Test for correlation between mean VAS score and the other sleep parameters at each post-operative time point (i.e.,: ten time points, from POST1 to POST10) were performed using Pearson’s method, after confirming the normal distribution of each values by the Kolmogorov-Smirnov test. Correlations were considered significant when *r*>0.3 and *p*<0.05. Statistical analysis was performed using GraphPad Prism (GraphPad Software, San Diego, CA, USA).

## Results

*N*=28 participants were screened for eligibility and met the inclusion criteria. A total of *n*=8 subjects dropped out: *n*=5 due to the non-adherence to the experimental procedures (i.e., not wearing the actigraphs for eleven consecutive nights or because the sleep diary was not correctly filled) and *n*=3 for technical problem during data acquisition. Therefore, *n*=20 completed the study with no adverse events related to surgery (11 males and 9 females; age: 64.0±10.39 years old; BMI: 29.1±3.85 kg*m^2^). In total, 14 patients underwent knee joint replacement (4 total and 10 unicompartmental) whereas six patients underwent total hip replacement. No significant inter-group differences (knee vs hip) were detected in sleep parameters.

### Sleep quality and quantity

**Fig. 2:**
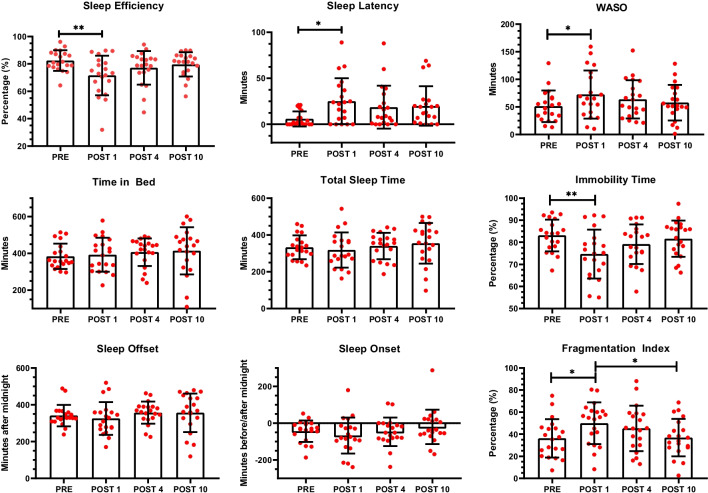
Individual data plots (*n*=20; in red) and mean±SD (black lines) of each of the nine sleep parameters (SE, SL, WASO, TB, TST, IT, SOn, SOff, and FI) evaluated by actigraphy in PRE, POST1, POST4, and POST10. Legend: *p<0.05; **p<0.01

Raw data and mean ± SD of each sleep parameter are shown in Fig. 2 while Table 1 shows mean ± SD, the results of RM-ANOVA and ES for significant outcomes. Actigraph parameters of sleep quantity (TB and TST) and timing (SOn and SOff) did not differ from PRE to POST10, during the hospitalization. On the contrary, SE (*p*=0.006; *η*^2^_p_=0.45, large), SL (*p*=0.039; *η*^2^_p_=0.37, large), WASO (*p*=0.042; *η*^2^_p_=0.34, large), IT (*p*=0.005; *η*^2^_p_=0.47, large), and FI (*p*=0.016; *η*^2^_p_=0.36, large) displayed significant differences. Specifically, SE and IT significantly decreased at POST1 compared to PRE by 10.8% (*p*=0.003; ES: 0.9, moderate) and 9.4% (*p*=0.005; ES: 0.86, moderate) respectively. SL increased by 18.7 min (+320%) at POST1 compared to PRE (*p*=0.046; ES: 0.70, moderate) and similarly WASO increased from 51.1 ± 28.4 min to 73.9 ± 46.7 min from PRE to POST1 (*p*=0.042; ES: 0.58, small). In addition, FI showed higher values in POST1 (51.2 ± 21.6 %) compared both to PRE (36.2 ± 17.4 %; *p*=0.034 and ES: 0.76, moderate) and POST10 (37.3 ± 17.3 %; *p*=0.047 and ES: 0.71, moderate) Overall, all sleep quality parameters (SE, SL, WASO, IT, and FI) showed a trend of constant improvement from POST1 to POST10.

**Table 1:** Subjects’ (*n*=20) actigraphy-based sleep parameters in PRE, POST1, POST4, and POST10

	PRE	POST1	POST4	POST10	RM-ANOVA	Partial eta-squared (*η*^2^_p_)	Tukey-Kramer post hoc test
Sleep efficiency (%)*	82.3 ± 7.5	71.5 ± 14.5	77.1 ± 12.2	79.5 ± 8.8	*p*=0.006	*F*_3,19_=5.29; *η*^2^_p_=0.45	PRE>POST1 (*p*=0.003)
Sleep latency (min)*	8.5 ± 13.2	27.2 ± 35.2	18.7 ± 23.3	21.0 ± 21.3	*p*=0.039	*F*_3,19_=3.78; *η*^2^_p_=0.37	PRE<POST1 (*p*=0.046)
Time in Bed *(min)*	383.7 ± 68.8	392.4 ± 92.3	406.5 ± 75.1	410.6 ± 131.0	ns	-	-
WASO (min)*	51.1 ± 28.4	73.9 ± 46.7	66.6 ± 44.0	59.3 ± 32.0	*p*=0.042	*F*_3,19_=3.32; *η*^2^_p_=0.34	PRE<POST1 (*p*=0.042)
Total sleep time *(min)*	332.6 ± 64.5	318.5 ± 95.9	339.8 ± 71.9	351.2 ± 112.1	ns	-	-
Immobility time *(%)*	83.0 ± 7.2	73.6 ± 13.5	78.6 ± 10.4	81.2 ± 8.2	*p*=0.005	*F*_3,19_=5.68; *η*^2^_p_=0.47	PRE>POST1 (*p*=0.005)
Sleep offset (min)*	341.0 ± 57.9	325.2 ± 88.8	357.0 ± 60.1	355.7 ± 107.4	ns	-	-
Sleep onset (min)*	-43.8 ± 58.5	-67.3± 98.2	-47.0± 77.9	-22.5 ± 95.4	ns	-	-
Fragmentation index *(%)*	36.2 ± 17.4	51.2 ± 21.6	45.2 ± 20.5	37.3 ± 17.3	*p*=0.016	*F*_3,19_=3.69; *η*^2^_p_=0.36	PRE<POST1 (*p*=0.034) POST1>POST4 (*p*=0.047)

Data are reported as mean ± SD. *Sleep efficiency, sleep latency, WASO, sleep onset, and sleep offset were not normally distributed and were subjected to the nonparametric Friedman test followed by the Dunn’s multiple comparisons. Abbreviations: WASO, wake after sleep onset; Ns, no significant differences; RM-ANOVA, repeated-measures analysis of variance.

PSQI mean scores significantly increased at POST4 compared to PRE (5.4±3.6 vs 8.1±3.8; *p*=0.006; ES: 0.72, moderate) highlighting a worsening of subjective sleep quality.

### Pain and sleep

As expected, VAS scores were significantly higher in the first day post-surgery (4.58 ± 2.46; *p*=0.0011 and ES: 1.40, large) compared to POST10 (1.68 ± 1.58) and in POST4 (3.85 ± 2.31) compared to POST10 (−2.18; *p*=0.0087 and ES: 1.09, moderate). During time, mean VAS showed significant negative correlations with mean SE (*r* = −0.71; *p*=0.021) and mean IT (*r* = −0.83; *p*=0.003) and a significant positive correlation with FI (*r* = 0.70; *p*=0.023), highlighting that high scores of pain were associated with lower overall sleep quality. No significant correlations were observed between mean VAS and the other sleep parameters during time.

### Clinical outcomes

FIM, TUG, 10MWT, and BI showed significant changes among the three time points (*p*<0.0001) and the values were always worse at POST4 compared both to PRE and POST10 for all clinical outcomes. In addition, FIM and TUG scores at POST10 (i.e., before discharge) were lower compared to pre-surgery values. Overall, the clinical and functional results showed a clear improvement from POST4 to POST10.

## Discussion

To the best of our knowledge, this is the first study evaluating changes in sleep parameters for eleven consecutive days in hospitalized patients undergoing hip and knee arthroplasty with an objective and non-invasive method, i.e., by actigraphy. Actigraphs are accurate and valid instruments to study sleep, if compared to the gold standard polysomnography (PSG), and they are also widely used both in research and clinical practice to monitor activity levels and rest-activity circadian rhythm in a practical way [33, 43]. The results of the present study showed that (1) sleep quantity and sleep timing parameters were stable during the entire hospitalization, indicating that patients started and ended to sleep at the same time and reached the same amount of sleep each night; (2) objective sleep quality parameters significantly worsened the first night after surgery compared to the pre-surgery night and, overall, these parameters showed a trend of constant improvement until discharge; (3) pain scores were at the highest level the first day after surgery and at the lowest level before discharge with a negative correlation between pain and sleep quality parameters during time; (4) the clinical outcomes followed an expected clinical path, and higher scores were observed immediately before discharge. Our initial hypotheses were all confirmed.

Having a normal sleep cycle is crucial to guarantee the normal function of humans’ psycho-physiological processes [43–45]. A physiological sleep-wake ultradian cycle should last about 90 min, showing four different stages (N1, N2, N3, and REM) and the sleep period should be seven to nine h per night [4, 46]. Experiencing sleep restriction (i.e., the partial reduction of sleep volume) or, even worst, sleep deprivation (i.e., the total absence of sleep) can have detrimental effects for almost every system of the human body and can lead to an increased risk for diabetes, obesity, immunodeficiency, cardiovascular disease, hormonal imbalance, pain developing, and mental health or mood disorders [22, 47–49]. For these reasons, it is easily understandable why having proper sleep is important in rehabilitation after surgery too [50]. Our study sample was composed of 20 patients undergoing knee and hip surgery and we analyzed their sleep characteristics starting from the night before surgery, coincident to the 1st night of hospitalization, up to the tenth day after surgery, before discharge. The day before surgery, at baseline, we observed that a large percentage of subjects did not obtain the National Sleep Foundation recommendations for sleep quality and quantity [4, 46]. In detail, 75% of subjects (15/20) were short-sleepers, with a total sleep time < six h, 55% (11/20) were poor-sleepers, with a sleep efficiency < 85%, and 75% (15/20) had a WASO > 30 min. Further, 55% of the study subjects had a pre-surgery PSQI score > 5, highlighting general subjective sleep problems. These results are in line with a previous study of Bjurstrom and colleagues where it was observed that 73.1% of patients waiting for total hip arthroplasty were classified as poor sleepers (PSQI > 5) the day before surgery [18]. In this context, it is important to highlight that pre-surgery poor sleep can negatively impact postoperative outcomes. A recent systematic review and meta-analysis by Varallo and colleagues [51] reported that preoperative sleep disturbances are predictors of post-surgery pain intensity and, similarly, it was observed that pre-surgery sleep disorders predicted the first 24-h postsurgical increase in opioid treatment [18]. However, in the present study, we did not observe higher postoperative pain levels or increased opioid treatment in those subjects that were classified as short- or poor-sleepers before surgery.

The main possible causes for disturbed sleep after surgery are related to inflammatory stress surgical response, discomfort, and postoperative pain [52, 53]. In our study, we observed a clear drop of sleep quality parameters the first night following surgery with a reduction of −10.8% in sleep efficiency, −9.4% in immobility time, and a +18.7 min, +22.8 min, and +15.0% increase in sleep latency, WASO, and fragmentation index, respectively. On the contrary, sleep quantity (TIB and TST) and sleep timing (SOn and SOff) parameters were not negatively influenced by surgery and remained stable until discharge. Previous studies examined sleep changes after orthopaedic surgery. Myoji et al. [14] studied changes in sleep-wake cycle and sleep quality through actigraphy 2 days before and 1 day after total hip arthroplasty and they reported a significant worsening in actual sleep time (−90 min), total sleep time (−18%), subjective sleep quality, measured by the Oguri–Shirakawa–Azumi Sleep Inventory, Middle-Aged and Aged Version, and an increase in the number of nocturnal awakenings and pain scores. Similarly, the night-time sleep, measured by PSG, of fast-track patients undergoing knee or hip arthroplasty significantly worsened on the first postoperative night compared to the night before surgery at home. In detail, REM sleep time decreased from 18.2 to 1.2%, wake time increased from 19.1 to 44.3%, and, in addition, overall sleep architecture returned to pre-admission levels on the 4th night after surgery [52]. The latter result is only partially in line with our study: we observed a trend of constant improvement in sleep quality parameters from the fourth night after surgery until discharge; however, sleep characteristics did not return to pre-surgery levels.

Different types of anaesthesia and postoperative analgesic regimens can have an influence on patient’s sleep and pain perception. We controlled for these potential confounders since our participants were all treated with the same standardized analgesic strategy: spinal anesthesia and multimodal opioid-sparing postoperative analgesia. Said that, it is important to consider that poor pain management can negatively impact patients’ physical recovery and it is known that higher levels of pain may lead to sleep problems and, conversely, that also increased sleep disturbances can trigger pain [54]. In this study, we observed similar trends for sleep quality and pain scores, with a negative correlation between them, highlighting that lower pain scores were associated with higher sleep quality during the course of the rehabilitation. However, the bidirectional interaction between sleep and pain is very complex and not fully understood [55] and the nature of our study does not allow to infer causality; we are not able to understand if the reduction of pain scores determined a direct increase in sleep quality or vice versa. In this context, future studies, with different study design and aims, should address this issue.

Some limitations need to be acknowledged. First, we were not able to collect real baseline data on subjects’ sleep before hospitalization. Participants were indeed recruited just 1 day before surgery and they came from outside Lombardy region so it was not possible to start the sleep monitoring in a “real-life” condition (i.e., while they were sleeping at home). Further, we considered as baseline value the sleep of the first day of hospitalization, before surgery, but we are aware that hospitalization itself, regardless of its cause, can harm and negatively impact patients’ sleep architecture due to noise, light exposure, shared bedroom environment, anxiety, and stress [56]. In addition, sleeping in a new bedroom environment typically has a negative impact on sleep during the first night. This condition, known as the “first night effect” (FNE), could have also contributed to worsen sleep parameters during the first phases of the hospitalization [57]. However, FNE has a complex pattern and it has not yet been deeply studied in elderly surgical population. Second, daytime napping behavior was not monitored even if recent studies showed that diurnal naps are advantageous interventions to enhance recovery process, increase mood and psychological state, and to counteract the negative effect of sleep restriction on physical outcomes [58]. Third, although the study population was balanced for gender (12 males and 10 females), the participants underwent three different kinds of surgery: total knee, unicompartmental knee, and total hip joint replacement. Nevertheless, the inter-group analysis did not highlight any difference in sleep or clinical outcomes according to the different kinds of surgery and the three rehabilitation protocols were extremely similar among them.

## Conclusion

Taken all together, the present study confirms the important impact of surgery on sleep parameters, especially on the first few post-operative days. On the other hand, sleep tends to improve overtime and goes back to pre-surgery parameters after around ten days. Many strategies and policy could eventually improve this result, such as managing more carefully lights and noises in the rehabilitation ward or accommodate patients to single rooms instead of double rooms. Our findings create an important baseline that should be considered when developing future interventional studies on sleep and musculoskeletal rehabilitation.

## Data Availability

The data presented in this study are openly available in Zenodo at https://zenodo.org/record/7920299#.ZFuO-3ZBzFg.

## References

[1] Vitale JA, Banfi G, Sias M, La Torre A (2019). Athletes’ rest-activity circadian rhythm differs in accordance with the sport discipline. Chronobiol Int.

[2] Haus E, Smolensky M (2006). Biological clocks and shift work: circadian dysregulation and potential long-term effects. Cancer Causes Control.

[3] Dattilo M, Antunes HKM, Medeiros A (2011). Sleep and muscle recovery: endocrinological and molecular basis for a new and promising hypothesis. Med Hypotheses.

[4] Hirshkowitz M, Whiton K, Albert SM (2015). National Sleep Foundation’s updated sleep duration recommendations: final report. Sleep Health.

[5] Van Dongen HPA, Maislin G, Mullington JM, Dinges DF (2003) The cumulative cost of additional wakefulness: dose-response effects on neurobehavioral functions and sleep physiology from chronic sleep restriction and total sleep deprivation. Sleep. 10.1093/sleep/26.2.117 10.1093/sleep/26.2.117 12683469

[6] Scott JPR, McNaughton LR, Polman RCJ (2006). Effects of sleep deprivation and exercise on cognitive, motor performance and mood. Physiol Behav.

[7] Knutson KL (2007). Impact of sleep and sleep loss on glucose homeostasis and appetite regulation. Sleep Med Clin.

[8] Spiegel K, Tasali E, Penev P, Van Cauter E (2004). Brief communication: sleep curtailment in healthy young men is associated with decreased leptin levels, elevated ghrelin levels, and increased hunger and appetite. Ann Intern Med.

[9] Vgontzas AN, Zoumakis E, Bixler EO (2004). Adverse effects of modest sleep restriction on sleepiness, performance, and inflammatory cytokines. J Clin Endocrinol Metabol.

[10] Katz JN, Arant KR, Loeser RF (2021). Diagnosis and treatment of hip and knee osteoarthritis: a review. JAMA.

[11] Ulivi M, Orlandini L, Vitale JA (2021). Direct superior approach versus posterolateral approach in total hip arthroplasty: a randomized controlled trial on early outcomes on gait, risk of fall, clinical and self-reported measurements. Acta Orthop.

[12] Marullo M, Vitale JA, Stucovitz E, Romagnoli S (2019). Simultaneous bilateral unicompartmental knee replacement improves gait parameters in patients with bilateral knee osteoarthritis. Knee.

[13] Romagnoli S, Vitale JA, Marullo M (2020). Outcomes of lateral unicompartmental knee arthroplasty in post-traumatic osteoarthritis, a retrospective comparative study. Int Orthop.

[14] Myoji Y, Fujita K, Mawatari M, Tabuchi Y (2015). Changes in sleep-wake rhythms, subjective sleep quality and pain among patients undergoing total hip arthroplasty. Int J Nurs Pract.

[15] Wylde V, Rooker J, Halliday L, Blom A (2011). Acute postoperative pain at rest after hip and knee arthroplasty: severity, sensory qualities and impact on sleep. Orthop Traumatol Surg Res.

[16] Fatah RN, Abdulrahman B (2020). A sleep disturbance after total knee arthroplasty. J Fam Med Prim Care.

[17] Cremeans-Smith JK, Millington K, Sledjeski E (2006). Sleep disruptions mediate the relationship between early postoperative pain and later functioning following total knee replacement surgery. J Behav Med.

[18] Bjurström MF, Irwin MR, Bodelsson M (2021). Preoperative sleep quality and adverse pain outcomes after total hip arthroplasty. Eur J Pain.

[19] Waterman F, Cisternas M, Korrer S, Wilson A (2021). Analysis of patient characteristics, health care costs by surgical venue, and opioid utilization for common orthopedic procedures in the United States. J Manag Care Spec Pharm.

[20] Frers A, Shaffer J, Edinger J, Wachholtz A (2021). The relationship between sleep and opioids in chronic pain patients. J Behav Med.

[21] Kjølhede P, Langström P, Nilsson P et al (2012) The impact of quality of sleep on recovery from fast-track abdominal hysterectomy. J Clin Sleep Med 8. 10.5664/jcsm.2032 10.5664/jcsm.2032 PMC3407258 22893770

[22] Cappuccio FP, D’Elia L, Strazzullo P, Miller MA (2010). Sleep duration and all-cause mortality: a systematic review and meta-analysis of prospective studies. Sleep.

[23] Vitale JA, Negrini F, Rebagliati G, Giacomelli L, Donzelli S, Banfi G (2019) Actigraphy-based sleep parameters and restactivity circadian rhythm in a young scoliotic patient treated with rigid bracing: a case study. Yale J Biol Med 92(2):205–212 PMC6585518 31249481

[24] Masaracchio M, Hanney WJ, Liu X (2017). Timing of rehabilitation on length of stay and cost in patients with hip or knee joint arthroplasty: a systematic review with meta-analysis. PloS One.

[25] Pennestrì F, Negrini F, Banfi G (2020). Rehabilitation after knee arthroplasty. An accelerated multidisciplinary approach. Recenti Prog Med.

[26] Vitale JA, Banfi G, Tivolesi V (2021). Rest-activity daily rhythm and physical activity levels after hip and knee joint replacement: the role of actigraphy in orthopedic clinical practice. Chronobiol Int.

[27] Fascio E, Vitale JA, Sirtori P (2022). Early virtual-reality-based home rehabilitation after total hip arthroplasty: a randomized controlled trial. J Clin Med.

[28] Manning BT, Kearns SM, Bohl DD (2017). Prospective assessment of sleep quality before and after primary total joint replacement. Orthopedics.

[29] von Elm E, Altman DG, Egger M (2014). The strengthening the reporting of observational studies in epidemiology (STROBE) statement: Guidelines for reporting observational studies. Int J Surg.

[30] Mollayeva T, Thurairajah P, Burton K (2016). The Pittsburgh sleep quality index as a screening tool for sleep dysfunction in clinical and non-clinical samples: a systematic review and meta-analysis. Sleep Med Rev.

[31] Jacquot F, Ait Mokhtar M, Sautet A (2013). The mini postero-postero-lateral mini incision in total hip arthroplasty. Int Orthop.

[32] Price AJ, Alvand A, Troelsen A (2018). Knee replacement. The Lancet.

[33] Ancoli-Israel S, Cole R, Alessi C (2003). The role of actigraphy in the study of sleep and circadian rhythms. Sleep.

[34] McCormack HM, Horne DJ, Sheather S (1988). Clinical applications of visual analogue scales: a critical review. Psychol Med.

[35] Jensen MP, Chen C, Brugger AM (2003). Interpretation of visual analog scale ratings and change scores: a reanalysis of two clinical trials of postoperative pain. J Pain.

[36] Peters DM, Fritz SL, Krotish DE (2013). Assessing the reliability and validity of a shorter walk test compared with the 10-Meter Walk Test for measurements of gait speed in healthy, older adults. J Geriatr Phys Ther.

[37] Podsiadlo D, Richardson S (1991). The timed “up & go”: a test of basic functional mobility for frail elderly persons. J Am Geriatr Soc.

[38] Dodds TA, Martin DP, Stolov WC, Deyo RA (1993). A validation of the Functional Independence Measurement and its performance among rehabilitation inpatients. Arch Phys Med Rehabil.

[39] Mahoney FI, Barthel DW (1965). Functional evaluation: the Barthel Index. Md State Med J.

[40] Castiglia SF, Galeoto G, Lauta A (2017). The culturally adapted Italian version of the Barthel Index (IcaBI): assessment of structural validity, inter-rater reliability and responsiveness to clinically relevant improvements in patients admitted to inpatient rehabilitation centers. Funct Neurol.

[41] Bakeman R (2005). Recommended effect size statistics for repeated measures designs. Behav Res Methods.

[42] Hopkins WG, Marshall SW, Batterham AM, Hanin J (2009). Progressive statistics for studies in sports medicine and exercise science. Med Sci Sports Exerc.

[43] Vitale JA, Perazzo P, Silingardi M (2020). Is disruption of sleep quality a consequence of severe Covid-19 infection? A case-series examination. Chronobiol Int.

[44] Shapiro CM, Flanigan MJ (1993) ABC of sleep disorders. Function of sleep. BMJ 306. 10.1136/bmj.306.6874.383 10.1136/bmj.306.6874.383 PMC1676416 8461688

[45] Briguglio M, Vitale JA, Galentino R et al (2020) Healthy eating, physical activity, and sleep hygiene (HEPAS) as the winning triad for sustaining physical and mental health in patients at risk for or with neuropsychiatric disorders: considerations for clinical practice. Neuropsychiatr Dis Treat 16. 10.2147/NDT.S229206 10.2147/NDT.S229206 PMC6955623 32021199

[46] Ohayon M, Wickwire EM, Hirshkowitz M (2017). National Sleep Foundation’s sleep quality recommendations: first report. Sleep Health.

[47] Filipas L, Ferioli D, Banfi G (2021). Single and combined effect of acute sleep restriction and mental fatigue on basketball free-throw performance. Int J Sports Physiol Perform.

[48] Vitale JA, Bonato M, Petrucci L (2021). Acute sleep restriction affects sport-specific but not athletic performance in junior tennis players. Int J Sports Physiol Perform.

[49] Kim TW, Jeong JH, Hong SC (2015, 2015) The impact of sleep and circadian disturbance on hormones and metabolism. Int J Endocrinol 591729 10.1155/2015/591729 PMC4377487 25861266

[50] Ellis BW, Dudley HAF (1976). Some aspects of sleep research in surgical stress. J Psychosom Res.

[51] Varallo G, Giusti EM, Manna C et al (2022) Sleep disturbances and sleep disorders as risk factors for chronic postsurgical pain: a systematic review and meta-analysis. Sleep Med Rev 63 10.1016/j.smrv.2022.101630 35594644

[52] Krenk L, Jennum P, Kehlet H (2012). Sleep disturbances after fast-track hip and knee arthroplasty. Br J Anaesth.

[53] Krenk L, Jennum P, Kehlet H (2014). Postoperative sleep disturbances after zolpidem treatment in fast-track hip and knee replacement. J Clin Sleep Med.

[54] Konjarski M, Murray G, Lee VV, Jackson ML (2018). Reciprocal relationships between daily sleep and mood: a systematic review of naturalistic prospective studies. Sleep Med Rev.

[55] Andersen ML, Araujo P, Frange C, Tufik S (2018). Sleep disturbance and pain: a tale of two common problems. Chest.

[56] Stewart NH, Arora VM (2018). Sleep in hospitalized older adults. Sleep Med Clin.

[57] Lorenzo JL, Barbanoj MJ (2002). Variability of sleep parameters across multiple laboratory sessions in healthy young subjects: the “very first night effect”. Psychophysiology.

[58] Lastella M, Halson SL, Vitale JA (2021). To nap or not to nap? A systematic review evaluating napping behavior in athletes and the impact on various measures of athletic performance. Nat Sci Sleep.

